# SafePsych: Improving patient safety by delivering high-impact simulation training on rare and complex scenarios in psychiatry

**DOI:** 10.1192/j.eurpsy.2023.710

**Published:** 2023-07-19

**Authors:** K. Tong, E. McMahon, B. Reid-McDermott, D. Byrne, A. M. Doherty

**Affiliations:** ^1^National Forensic Mental Health Service, Central Mental Hospital, Dundrum, Dublin; ^2^Department of Psychiatry, University Hospital Galway; ^3^Irish Centre for Applied Patient Safety and Simulation, National University of Ireland, Galway; ^4^Department of Psychiatry, School of Medicine & Medical Science, University College Dublin; ^5^Department of Psychiatry, Mater Misericordiae University Hospital, Dublin, Ireland

## Abstract

**Introduction:**

Simulation-based training is a practical medical education tool to develop health professionals’ knowledge and experience in a low risk, realistic clinical setting. It trains clinicians to recognise and manage rare and complex clinical scenarios without compromising patient safety. Despite an evidence base demonstrating simulation to be an effective medical education tool, it is not commonly used in postgraduate psychiatry training as it is in other medical specialties.

**Objectives:**

This project outlines the development and effectiveness of a hybrid-virtual simulation-based workshop designed to improve patient care by improving clinical skills of non-consultant hospital doctors (NCHDs) in detecting and managing rare and complex psychiatric emergencies.

**Methods:**

Three clinical vignettes based on near-miss clinical scenarios in psychiatry were developed by a multidisciplinary team of doctors and nurses in psychiatry, and experts in simulation-based medical education. The workshop, ‘SafePsych’ was delivered in a simulation laboratory, while being captured on camera and broadcasted via Zoom video-conferencing platform to observers. Debriefing followed each clinical scenario. Participants completed pre- and post-workshop questionnaires to evaluate clinical knowledge of the scenarios in the training programme.

**Results:**

The workshop was attended by consultants (n=12), NCHDs in psychiatry and emergency medicine (n=19), and psychiatric nurses (n=5). In the psychiatry NCHD group, test scores significantly improved following the workshop (p<0.001). There were significant improvements in the test scores with a mean difference of 2.56 (SD 1.58, p<0.001). Feedback from participants and observers was positive, with constructive appraisals to improve the virtual element of the workshop.

**Conclusions:**

Simulation-based training is effective in teaching high risk, rare complex psychiatric cases to psychiatry NCHDs. Further exploration of the learning needs of nursing staff is required. Future workshop delivery is feasible in the COVID-19 environment and beyond, using a virtual element to meet social distancing requirements while enhancing the reach of the training.

**Disclosure of Interest:**

None Declared

